# Brain-Derived Neurotrophic Factor (BDNF) Concentration Levels in Cerebrospinal Fluid and Plasma in Patients With Glioblastoma: A Prospective, Observational, Controlled Study

**DOI:** 10.7759/cureus.48237

**Published:** 2023-11-03

**Authors:** Katarzyna Wójtowicz, Katarzyna Czarzasta, Lukasz Przepiorka, Sławomir Kujawski, Agnieszka Cudnoch-Jedrzejewska, Andrzej Marchel, Przemysław Kunert

**Affiliations:** 1 Department of Neurosurgery, Medical University of Warsaw, Warsaw, POL; 2 Department of Experimental and Clinical Physiology, Laboratory of Centre for Preclinical Research, Medical University of Warsaw, Warsaw, POL; 3 Department of Exercise Physiology and Functional Anatomy, Ludwik Rydygier Collegium Medicum in Bydgoszcz, Nicolaus Copernicus University in Toruń, Bydgoszcz, POL

**Keywords:** brain-derived neurotrophic factor (bdnf), elisa, cerebrospinal fluid (csf), marker, glioblastoma

## Abstract

Objective

Glioblastomas (GBMs) are among the most frequent and most malignant of untreatable brain tumors. A GBM marker could accelerate diagnosis and facilitate therapeutic monitoring. This prospective, observational, controlled study compared brain-derived neurotrophic factor (BDNF) levels in cerebrospinal fluid (CSF) and plasma between patients with GBM and a control group.

Materials and methods

Patients in the observational group underwent elective GBM resection (n=24, 55.8%). Control patients (n=19, 44.2%) had elective brain surgery for an unrelated, non-neoplastic, non-traumatic pathology. We measured BDNF levels in tumors, CSF, and plasma with enzyme-linked immunosorbent assay (ELISA). Peripheral blood and CSF samples were collected before surgery, and tumors were sampled intraoperatively. We analyzed correlations between BDNF levels and patient sex, age, seizures, smoking, diabetes mellitus (DM), and the use of selected antiepileptic drug (AED) and antihypertensive drug groups.

Results

The mean CSF BDNF concentration was significantly lower in patients with GBM (6.5 pg/mL) than in controls (11.48 pg/mL) (p=0.002). Similarly, the mean plasma BDNF concentration was significantly lower in patients with GBM (288.59 pg/mL) than in controls (574.06 pg/mL) (p=0.0005). None of the examined factors influenced CSF, plasma, or tumor tissue BDNF concentrations (p>0.05).

Conclusion

Plasma and CSF BDNF levels were significantly lower in adults with GBM than in controls. Thus, CSF and plasma BDNF levels may aid in GBM diagnoses. Further prospective studies are required.

## Introduction

Glioblastoma (GBM) is among the most formidable of cancer diagnoses. It has a relatively high prevalence, it is the second most common primary brain tumor, and it has a poor prognosis, with a one-year survival of 35.7% [1]. Thus, a prompt diagnosis and immediate treatment are required to improve the quality of life and extend life expectancy. Accordingly, many researchers have searched for an easily accessible GBM marker [2].

GBM markers could provide benefits for both diagnosis and therapeutic monitoring. For example, biomarkers are essential when a suspected GBM cannot be biopsied due to a deep or eloquent location or when a patient’s condition does not allow surgery.

Brain-derived neurotrophic factor (BDNF), a member of the neurotrophin family, is involved in several processes related to brain plasticity. BDNF levels are typically reduced in many neurodegenerative diseases and cognitive impairments [3]. BDNF is expressed by astrocytes, where it promotes proliferation and survival [4].

The present study aimed to compare BDNF levels in cerebrospinal fluid (CSF) and plasma between patients with GBM and control patients who underwent brain surgery for an unrelated, non-neoplastic, non-traumatic pathology.

## Materials and methods

Study design

This prospective, observational, controlled study was approved by the Bioethics Committee of the Medical University of Warsaw (approval number: KB/193/2016). We included all patients with a suspicion of a GBM, based on radiological findings and clinical presentation, who were qualified for elective surgical resection between 2016 and 2018 (observational group). A control group comprised patients who underwent elective brain surgery for unrelated conditions, including microsurgical clipping of unruptured intracranial aneurysms (UIAs) or another, non-neoplastic, non-traumatic pathology. Written consent was obtained from all participants.

**Figure 1:**
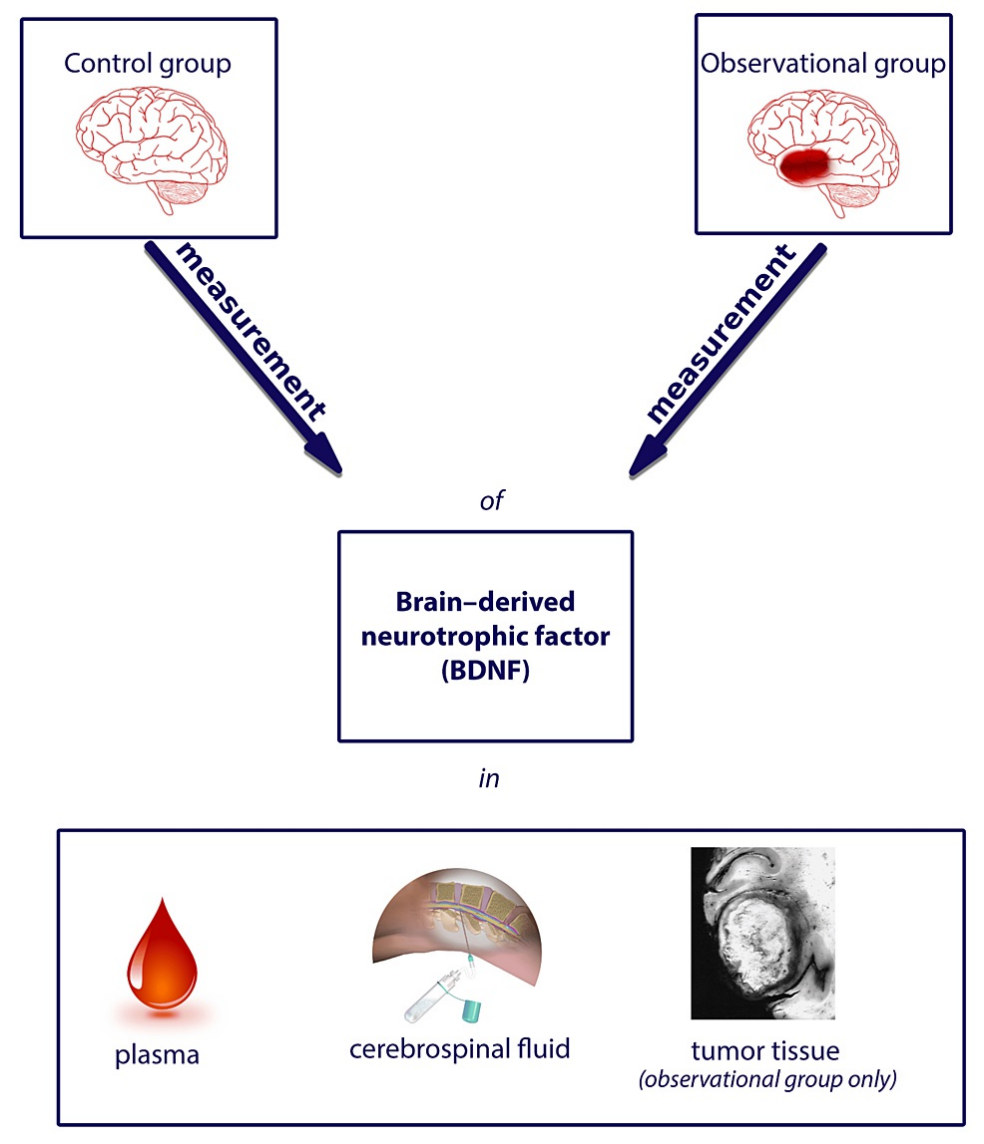
Study design BDNF levels in plasma and CSF were measured in the observational and control groups and in tumor samples in the observational group. The lumbar puncture image was adapted from Blausen.com staff (2014), “Medical gallery of Blausen Medical 2014,” WikiJournal of Medicine 1 (2), DOI: 10.15347/wjm/2014.010, ISSN 2002-4436, published under the Creative Commons Attribution 3.0 Unported License. BDNF: brain-derived neurotrophic factor, CSF: cerebrospinal fluid

In both groups, CSF and peripheral blood samples were examined, and in the observational group, tumor tissues were evaluated (Figure 1). Peripheral blood and CSF (via lumbar puncture) were collected before surgery, and tumor samples were collected intraoperatively. Plasma was separated within two hours from acquisition. Plasma, CSF, and tumor samples were stored at -70°C until use.

Peritumoral edema was assessed based on preoperative MRI scans, according to the Steinhoff scale [5]. After an integrated diagnosis, in accordance with the 2016 WHO Classification of Tumors of the Central Nervous System [6], patients with tumors other than GBM were excluded from the observational group.

BDNF assessments

Fragments of tumor tissue (100 mg) were homogenized in 500 µl phosphate-buffered saline (PBS), pH 7.4 (Merck KGaA, Darmstadt, Germany) supplemented with peptidase inhibitors, leupeptin, and aprotinin (Halt^TM^ Protease and Phosphatase Inhibitor Single-Use Cocktail, EDTA-Free, Thermo Fisher, Waltham, MA). We checked BDNF levels in tumor, CSF, and plasma samples with an enzyme-linked immunosorbent assay (ELISA) (Quantikine Human BDNF Elisa 96TST, DBD00, R&D Systems, Minneapolis, MN), according to manufacturer instructions. Protein standards and samples of plasma, cerebrospinal fluid, or homogenized tumor tissue were applied to a 96-well plate filled with Assay Diluent RD1S. Then, after two hours of incubation at room temperature, human free BDNF conjugate was added to all wells with standards and samples. After one hour of incubation, the liquid was removed, and the plate was washed three times in a wash buffer. Substrate solution assay was then applied to all wells of the plate. The reactions were stopped after 30 minutes by the addition of a stop solution assay. The result was read within 30 minutes of adding the stop solution using a microplate reader (iMark, Bio-Rad, Hercules, CA) set to 450 nm. Four parameter logistic (4-PL) protein standard curve fit was used to maintain the BDNF result in the examined tissues. BDNF concentrations in CSF and plasma are expressed in pg/mL, and BDNF levels in tumors are expressed in ng/g of tissue.

Statistical analysis

We performed the Shapiro-Wilk test and visually inspected histograms to test the assumption of data normality. We performed Levene’s test to assess the equality of variances. Between-group differences (control versus observational) were evaluated using the Mann-Whitney U or independent T-tests, depending on the assumptions met. We assessed correlations between quantitative variables with Spearman’s rank correlation coefficients. We examined associations between qualitative variables using the chi-squared test (https://www.icalcu.com/stat/chisqtest.html). All analyses were performed with a signiﬁcance level of α=0.05. Data in the main text are presented as the mean (range and median). Violin plots were created using Orange software (version 3.31.1) (University of Ljubljana, Slovenia) [7]. We performed statistical analysis with Statistica v.13.1. (StatSoft, Inc., Tulsa, OK).

## Results

Patients

The observational group included 24 patients (seven females and 17 males) (mean age: 59.46 years, range: 31-77 years). The control group included 19 patients (16 females and three males) (mean age: 59.74 years, range: 47-73 years). Table 1 presents the clinical characteristics of both study groups, including sex, age, seizures, smoking, diabetes mellitus (DM), and the use of the following selected drug groups: angiotensin-converting enzyme inhibitors (ACE-Is), angiotensin II receptor blockers (ARBs), and antiepileptic drugs (AEDs).

**Table 1:** Characteristics of surgical patients with GBM (observational group) compared to surgical patients with non-neoplastic, non-traumatic brain pathology (control group) Values are the number (%) or median (range), as indicated. ACA: anterior cerebral artery, ACE-I: angiotensin-converting enzyme inhibitor, ACoA: anterior communicating artery, AED: antiepileptic drug, ARB: angiotensin II receptor blocker, GBM: glioblastoma, ICA-PCoA: internal cerebral artery-posterior communicating artery, MCA: middle cerebral artery, N/A: not applicable, UIA: unruptured intracranial aneurysm

Characteristic	Control group		Observational group		p-value
Patients	19 (44.2%)		24 (55.8%)		0.44
Sex					0.0003
Male	3 (15.8%)		17 (70.8%)		
Female	16 (84.2%)		7 (29.2%)		
Age (years)	61 (47-73)		64 (31-77)		0.59
Diagnosis	Hemifacial spasm	1 (5.2%)	GBM region		N/A
	Trigeminal neuralgia	1 (5.2%)	* *Frontal	8 (33.3%)	
	Hydrocephalus	1 (5.2%)	* *Parietal	2 (8.3%)	
	UIA	16 (84.2%)	* *Temporal	12 (50%)	
	ACA	1 (6.25%)	Multiple lobes	2 (8.3%)	
	ACoA	1 (6.25%)	Tumor volume (cm^3^)	22 (3-85)	
	ICA-PCoA	2 (12.5%)	IDH mutant		
	* *MCA	11 (68.75%)	Yes	3 (12.5%)	
	MCA+ACoA	1 (6.25%)	No	17 (70.8%)	
			No data	4 (16.7%)	
			1p/19q codeletion		
			Yes	0 (0%)	
			No	24 (100%)	
Side					0.23
Right	10 (52.6%)		13 (54.2%)		
Left	6 (31.6%)		11 (45.8%)		
Both	1 (5.3%)		0 (0%)		
N/A	2 (10.5%)		0 (0%)		
Epileptic seizures					0.003
Yes	0 (0%)		9 (37.5%)		
No	19 (100%)		15 (62.5%)		
Reason for diagnosis					0.001
Headache	6 (31.6%)		4 (16.7%)		
Epileptic seizure	0 (0%)		7 (29.2%)		
Cognitive impairment	1 (5.3%)		5 (20.8%)		
Speech disorder	0 (0%)		5 (20.8%)		
Other neurological deficit	5 (26.3%)		2 (8.3%)		
Other	7 (36.8%)		1 (4.2%)		
Smoking					0.15
Yes	4 (21%)		3 (12.5%)		
No	15 (79%)		17 (70.8%)		
No data	0 (0%)		4 (16.7%)		
Chronic diseases					0.23
Yes	17 (89.5%)		18 (75%)		
No	2 (10.5%)		6 (25%)		
Other diseases					0.29
Yes	8 (42.1%)		14 (58.3%)		
No	11 (57.9%)		10 (41.7%)		
Hypertension					0.44
Yes	14 (73.7%)		15 (62.5%)		
No	5 (26.3%)		9 (37.5%)		
Diabetes					0.94
Yes	3 (15.8%)		4 (16.7%)		
No	16 (84.2%)		20 (83.3%)		
Diabetes treatment					0.55
Metformin	2 (10.5%)		3 (12.5%)		
Insulin	0 (0%)		1 (4.2%)		
Other	1 (5.3%)		0 (0%)		
None	16 (84.2%)		20 (83.3%)		
ACE-I					0.04
Yes	2 (10.5%)		9 (37.5%)		
No	17 (89.5%)		15 (62.5%)		
ARB					0.13
Yes	6 (31.6%)		3 (12.5%)		
No	13 (68.4%)		21 (87.5%)		
AED					0.08
Carbamazepine	1 (5.3%)		2 (8.3%)		
Valproic acid	0 (0%)		3 (12.5%)		
Levetiracetam	0 (0%)		4 (16.7%)		
Multidrug	0 (0%)		1 (4.2%)		
None	18 (94.7%)		14 (58.3%)		
Other medical treatment					0.46
Yes	16 (84.2%)		18 (75%)		
No	3 (15.8%)		6 (25%)		

BDNF concentrations in CSF and plasma

**Figure 2:**
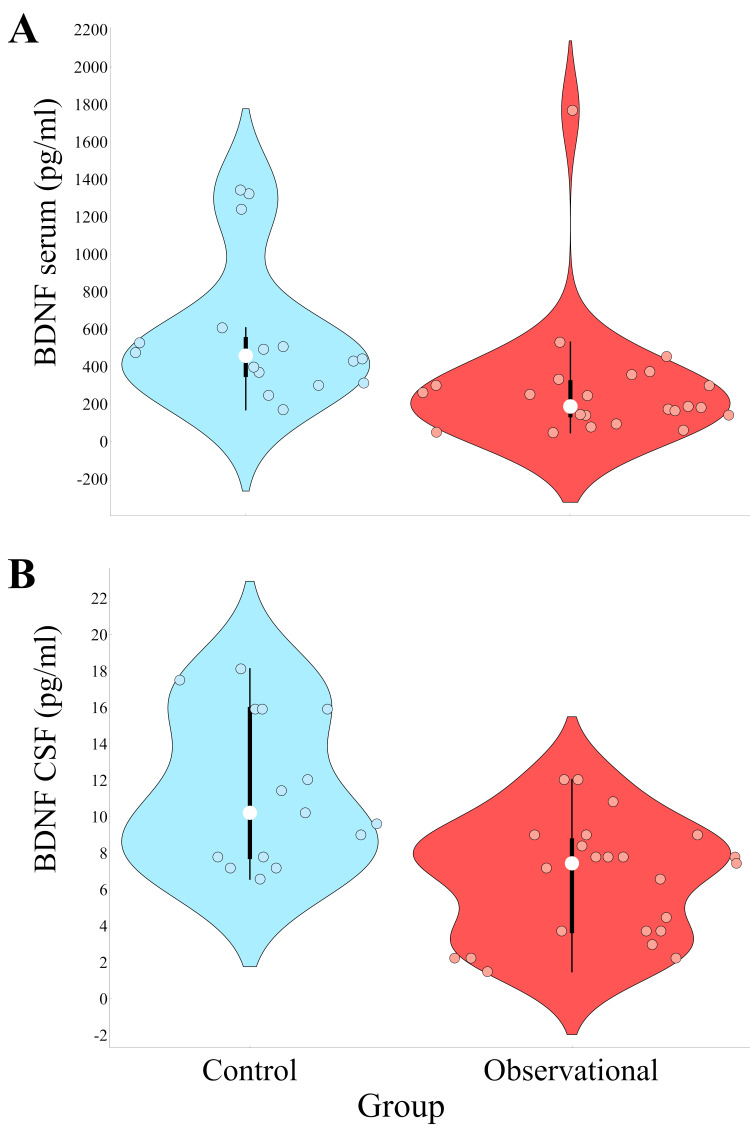
Differences in BDNF levels in CSF and plasma between observational (glioblastoma) and control (aneurysm) groups White dots inside the box plots denote the median value; the lower and upper sides of boxes denote the 25th and 75th percentiles, respectively; and the minimal and maximal values are denoted by the height of the vertical line. BDNF: brain-derived neurotrophic factor, CSF: cerebrospinal fluid

CSF BDNF concentrations were significantly lower in patients with GBM than in the control group (6.5 pg/mL versus 11.48 pg/mL) (p=0.002) (Figure 2A). Likewise, in plasma, BDNF concentrations were notably lower in GBM patients when compared to the control group (mean: 288.59 pg/mL versus 574.06 pg/mL) (p=0.0005) (Figure 2B).

BDNF levels in tumor tissue

Within tumor tissue, the average BDNF level was 14.23 ng/g tissue, with a range spanning from 1.75 to 54.55 ng/g and a median value of 6.01 ng/g.

Correlations between BDNF levels and examined factors

When examining the influence of IDH1 mutation status, there were no significant differences in mean CSF BDNF levels between patients with GBM who had the IDH-mutant or IDH-wildtype variant (5.59 pg/mL versus 6.4 pg/mL) (p=1). Similarly, for plasma BDNF levels, there were no significant differences between the two groups (246.67 pg/mL versus 263.16 pg/mL) (p=0.86).

The mean tumor tissue BDNF levels were 8.47 ng/g in patients with GBM IDH-mutant and 23.93 ng/g in patients with GBM IDH-wildtype (p=0.55). Thus, the IDH1 mutation had no effect on the BDNF concentrations in CSF, plasma, or tumor tissue. Likewise, other examined factors (such as age, cigarette smoking, ACE-I use, ARB use, seizures, and diabetes mellitus) had no significant relationship with BDNF levels in plasma (Table 2) or CSF (Table 3).

**Table 2:** Relationship between CSF BDNF levels and select potentially influential factors Values are the median (minimum-maximum), as indicated. ACE-I: angiotensin-converting enzyme inhibitor, AED: antiepileptic drug, ARB: angiotensin II receptor blocker, BDNF: brain-derived neurotrophic factor, CSF: cerebrospinal fluid, DM-2: diabetes mellitus type 2

Factor	Level	CSF BDNF level (pg/mL)	p-value
Smoking status	Yes	8.40 (2.23-15.91)	0.65
	No	7.79 (1.49-18.12)	
ACE-I	Yes	7.79 (1.49-15.91)	0.75
	No	7.79 (2.23-18.12)	
ARB	Yes	8.70 (7.79-18.12)	0.63
	No	7.79 (1.49-17.51)	
Epilepsy	Yes	7.18 (2.23-18.12)	0.053
	No	8.70 (1.49-17.51)	
DM-2	Yes	8.70 (2.23-10.83)	0.22
	No	7.79 (1.49-18.12)	

**Table 3:** Relationship between plasma BDNF levels and select potentially influential factors Values are the median (minimum-maximum), as indicated. ACE-I: angiotensin-converting enzyme inhibitor, AED: antiepileptic drug, ARB: angiotensin II receptor blocker, BDNF: brain-derived neurotrophic factor, DM-2: diabetes mellitus type 2

Factor	Level	Plasma BDNF (pg/mL)	p-value
Smoking status	Yes	312.35 (49.85-1323.11)	0.93
	No	300.00 (47.48-1343.24)	
ACE-I	Yes	298.55 (47.48-1768.83)	0.84
	No	332.61 (49.85-1343.24)	
ARB	Yes	369.88 (77.79-607.29)	0.12
	No	298.55 (47.48-1768.83)	
Epilepsy	Yes	204.98 (49.85-530.49)	0.10
	No	363.72 (47.48-1768.83)	
DM-2	Yes	432.28 (144.36-607.29)	1
	No	299.28 (47.48-1768.83)	

Correlations between BDNF levels

Moreover, we found no significant correlation between CSF and plasma BDNF concentrations in either group (rho=−0.04 (correlation coefficient), p=0.9, in the control group and rho=−0.2, p=0.38, in the observational group). Additionally, in the observational group, BDNF concentrations in tumor tissues did not correlate significantly with CSF concentrations (rho=0.36, p=0.11).

**Figure 3:**
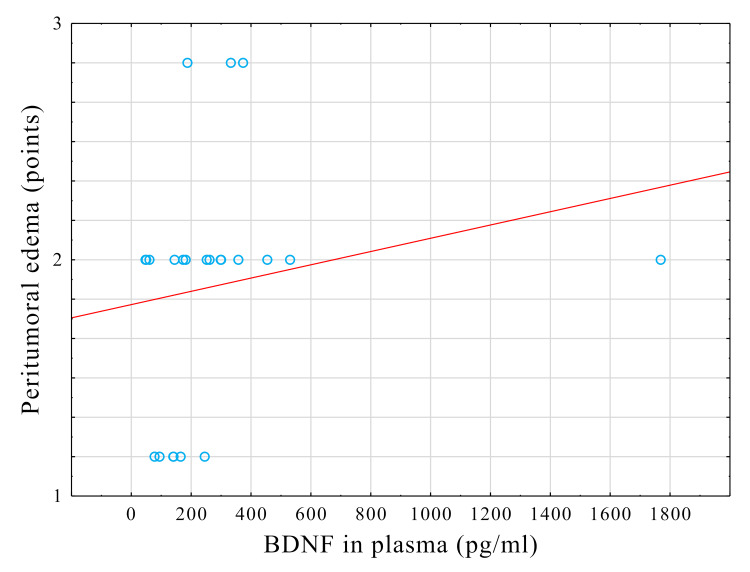
Scatterplot showing the relationship between BDNF in plasma and the degree of peritumoral edema Points: ratings according to the Steinhoff scale, based on preoperative MRI assessments BDNF: brain-derived neurotrophic factor, MRI: magnetic resonance imaging

We found no significant correlation between plasma BDNF and tumor size (rho=-0.04, p=0.8) or between CFS BDNF and tumor size (rho=-0.14, p=0.53). Furthermore, peritumoral edema was not significantly related to the CSF BDNF (rho=-0.21, p=0.34). However, peritumoral edema was positively correlated with plasma BDNF (rho=0.45, p=0.03) (Figure 3).

## Discussion

BDNF action is mediated by tropomyosin receptor kinase B, whose expression in gliomas may promote tumor growth and progression toward malignancy [8]. To investigate BDNF in clinical conditions, we assessed whether BDNF levels in CSF (which is in direct contact with tumor tissues) could be used as a potential GBM marker. We reasoned that CSF can be easily collected, regardless of a patient’s general condition; thus, it is a feasible material for analysis. Our results showed that the mean CSF and plasma BDNF concentrations were significantly lower in patients with GBM compared to the control group. Our findings contrasted with previous in vitro studies, which showed that BDNF expression was upregulated in human glioma tissues [9,10] compared to normal brain tissues [11]. For example, Xiong et al. found that BDNF inhibited C6 glioma cell apoptosis and increased cell growth and migration [10]. Previous in vivo studies have extensively explored BDNF effects in different diseases, including schizophrenia [12-14] and depression [15-17].

A growing number of in vitro studies have reported that BDNF levels were increased in GBMs. Those findings suggested a promising hypothesis for a potential GBM marker. However, no real-life conclusions had been drawn from those studies. Indeed, a recent review by Jones et al. on circulating glioma biomarkers did not even mention BDNF [18].

Chiaretti et al. (2004) found that, among children with brain tumors (i.e., astrocytomas and ependymomas), CSF BDNF levels were significantly higher compared to a control group [19]. To the best of our knowledge, no study has been published subsequent to that interesting result. Thus, in the present study, we hypothesized that the CSF BDNF concentration would be increased in patients with GBMs. However, we found the opposite result. The discrepancy in our findings compared to Chiaretti et al. likely stems from substantial differences in patient populations and tumor types. Chiaretti et al. evaluated a pediatric cohort with “low-grade gliomas,” which generally exhibit less invasive behavior than the highly aggressive GBMs targeted in our study [19]. Additionally, our research encompassed adult patients, broadening the age spectrum considerably. These variances in age groups and tumor types inherently introduce biases, making direct comparisons challenging. Moreover, another study (published in Chinese) by Yan et al. found that BDNF expression was greatly upregulated in human glioma tissue [20]. However, they tested gliomas of different WHO grades.

Our results suggested that, due to the infiltrative nature of GBMs, the tumor had a global effect on the brain. In that respect, it resembled a neurodegenerative disease associated with a reduction in BDNF. The infiltrative nature of GBM was highlighted by the lack of a relationship between the tumor volume and the BDNF concentration.

We found a correlation between plasma BDNF levels and peritumoral edema. However, because the effect size of this monotonic relationship could be considered moderate [21], that result was recognized as clinically insignificant, and hence, it was disregarded.

We found no correlation between CSF and plasma BDNF concentrations in either group. Furthermore, we found no correlation between tumor tissue and CSF BDNF concentrations in the observational group. In contrast, other studies have reported correlations between BDNF levels in different tissues. For example, Klein et al. suggested that blood and plasma BDNF levels were correlated with brain-tissue BDNF levels [22]. Pillai et al. observed a significant positive relationship between plasma and CSF BDNF levels in drug-naive patients with first-episode psychoses [23].

Our results showed that the IDH1 mutation had no effect on BDNF concentrations in CSF, plasma, or tumor tissues. Moreover, none of the other examined factors influenced the BDNF concentrations in plasma or CSF. In contrast, a previous study performed a weighted co-expression network analysis and showed that IDH1 was correlated with the expression of 10 hub genes, including BDNF [24]. Moreover, Lommatzsch et al. showed that plasma BDNF concentrations were negatively correlated with age and weight, but they observed no differences in plasma BDNF concentrations between the sexes [25].

Due to the significantly lower CSF and plasma BDNF levels in the study group, compared to controls, we expected that tumor size and peritumoral edema would be related to CSF and plasma BDNF levels. However, those relationships were not observed.

Some studies have reported relationships between BDNF and the renin-angiotensin system blockers, ACE-I, and ARB [26-28]. Accordingly, we included ACE-I and ARB in the present study. Table 1 shows that the studied groups differed in ACE-I use (10.5% and 37.5% used ACE-I in the control and observational groups, respectively). Nevertheless, in our cohort, ACE-I did not influence plasma or CSF BDNF levels. Similarly, Demir et al. found no difference in plasma BDNF levels among patients who did or did not use ACE-I, ARB, and other antihypertensive drugs [29].

We noted a difference in the presence of epileptic seizures between the study groups. However, we believe the difference was due to a selection bias. The control group consisted mostly of patients with UIAs, which rarely cause epileptic seizures, unlike GBMs.

Study limitations

Our study had some limitations. First, we included a small number of patients. Second, our analyses were performed with ELISA tests. According to Ou et al., ELISAs may not be sufficiently sensitive for accurately detecting CSF BDNF levels in patients with neurological disorders [30]. Furthermore, patients in the observational and control groups were demographically different (inverse male-to-female ratios). Additionally, our control group consisted mostly of patients with UIAs who required treatment. Finally, our patients were diagnosed according to the CNS WHO 2016 classification, which is no longer in use.

## Conclusions

This study showed that adults diagnosed with GBM had significantly lower BDNF levels in plasma and CSF compared to the control group. Additionally, the study provides insights into the relationship between BDNF and various clinical factors. Furthermore, our study suggests that IDH1 mutation does not play a significant role in modulating BDNF levels in GBM. These findings contribute to our understanding of BDNF in the context of GBM and its potential implications for the disease.

Our findings suggested that BDNF levels in CSF and plasma may aid in determining a GBM diagnosis. Further prospective studies are required to draw more conclusions and develop guidelines.
